# Links between Genetic Groups, Indole Alkaloid Profiles and Ecology within the Grass-Parasitic *Claviceps purpurea* Species Complex

**DOI:** 10.3390/toxins7051431

**Published:** 2015-04-28

**Authors:** Mariell Negård, Silvio Uhlig, Håvard Kauserud, Tom Andersen, Klaus Høiland, Trude Vrålstad

**Affiliations:** 1 Norwegian Veterinary Institute, P.O. Box 750 Sentrum, 0106 Oslo, Norway; E-Mails: marne11235@gmail.com (M.N.); silvio.uhlig@stami.no (S.U.); 2 Department of Biosciences, University of Oslo, P.O. Box 1066 Blindern, 0316 Oslo, Norway; E-Mails: havard.kauserud@ibv.uio.no (H.K.); tom.andersen@ibv.uio.no (T.A.); klaus.hoiland@ibv.uio.no (K.H.); 3 Department of the Chemical and Biological Working Environment, National Institute of Occupational Health, P.O. Box 8149 Dep, 0033 Oslo, Norway

**Keywords:** ergot fungus, ergot alkaloids, indole-diterpenes, nuclear rDNA, β-tubulin gene, haplotype network, speciation

## Abstract

The grass parasitic fungus *Claviceps purpurea* *sensu lato* produces sclerotia with toxic indole alkaloids. It constitutes several genetic groups with divergent habitat preferences that recently were delimited into separate proposed species. We aimed to 1) analyze genetic variation of *C. purpurea sensu lato* in Norway, 2) characterize the associated indole alkaloid profiles, and 3) explore relationships between genetics, alkaloid chemistry and ecology. Approximately 600 sclerotia from 14 different grass species were subjected to various analyses including DNA sequencing and HPLC-MS. Molecular results, supported by chemical and ecological data, revealed one new genetic group (G4) in addition to two of the three known; G1 (*C. purpurea sensu stricto*) and G2 (*C. humidiphila*). G3 (*C. spartinae*) was not found. G4, which was apparently con-specific with the recently described *C. arundinis* sp. nov, was predominantly found in very wet habitats on *Molinia caerulea* and infrequently in saline habitats on *Leymus arenarius*. Its indole-diterpene profile resembled G2, while its ergot alkaloid profile differed from G2 in high amounts of ergosedmam. In contrast to G1, indole-diterpenes were consistently present in G2 and G4. Our study supports and complements the newly proposed species delimitation of the *C. purpurea* complex, but challenges some species characteristics including host spectrum, habitat preferences and sclerotial floating ability.

## 1. Introduction

The ergot fungus *Claviceps purpurea* is a phytopathogenic ascomycete that parasitizes more than 400 species in the grass family (Poaceae) including wild and cultivated pasture- and forage grasses and common cereals [1,2]. The infection is located to the ovaries of the host plant, resulting in a sclerotium (called an ergot) that replaces the seed grain. The sclerotia are resting- and overwintering structures containing a wide variety of alkaloids (Figure S1). These alkaloids, comprising 0.5%–2% of the total sclerotium weight [3], target the central and peripheral nervous systems of invertebrate and vertebrate animals, leading to toxicoses and behavioural changes which reduce herbivory and thus enhance protection of the ergot sclerotia [4]. *Claviceps purpurea* is primarily known for its production of ergot alkaloids, which have severe effects on the nervous system and smooth muscles [5,6]. Ergotism, the disease caused by some of the ergot alkaloids found in sclerotia, was a major concern during the Middle Ages when epidemic outbreaks occurred regularly and lead to painful death for tens of thousands of people [4,7,8]. In modern time, outbreaks of ergotism have been sporadically reported from India and Africa [4], but in general the problem is now rare among humans due to modern agricultural practices. However, ergotism persists in the wildlife and sometimes among livestock [9,10,11]. In Norway, especially moose (*Alces alces*) are regularly observed with visible signs of ergotism [12]. The ergot alkaloids are likely the most important group of bioactive compounds produced by several *Claviceps* spp with activities that have widely been exploited by the pharmaceutical industry [8]. For example, the world’s total production of ergot alkaloids was in 2010 about 20,000 kg, and the use of these substances involve anti-migraine drugs, uterotonics, prolactin inhibitors and anti-Parkinson agents [4,8]. Similar compounds are also produced within other fungal genera (e.g. *Penicillium* and *Aspergillius*), and it was long believed that plant species in the family Convolvulaceae also formed ergot alkaloids [7,13]. However, it has recently been demonstrated that plants of the family Convolvulaceae form close associations with ergoline alkaloid producing *Periglandula* fungi within the Clavicipitaceae [14,15].

Another group of alkaloids, the indole-diterpenes, are bioactive compounds that exhibit mammalian and insect toxicity through activation of various ion channels [4]. Known symptoms of indole-diterpene poisoning in livestock include ataxia and sustained tremors [4,16], and several analogues are known for the staggers/tremors syndrome they can produce in mammals through neurological disturbances [4,17,18,19]. The indole-diterpenes are primarily known from and studied in species of *Claviceps* other than *C. purpurea* as well as from the closely related cool-season grass endophytes (*Epichloë* or *Neotyphodium* species) of the Clavicipitaceae [4]. However, for *C. purpurea* indole-diterpenes are largely unexplored. Many analyzed strains do not produce these compounds, and genome sequence analysis of one *C. purpurea* strain does not predict the production of indole diterpene end-products in *C. purpurea* [4]. Nevertheless, recent results suggest that one of the genetic groups of *C. purpurea* (c.f. G2, see below) does produce indole-diterpenes although their chemical structures remain to be characterized and might be new to science [20]. 

Although *C. purpurea* is a cosmopolitan fungus that infects a broad variety of grass species, it comprises multiple genetic lineages that seem to reflect ecological differentiation. Three main genetic groups (often referred to as “genotype” G1, G2 and G3) have been recognized on the basis of RAPD [21] and DNA sequencing of among others ITS and a β-tubulin gene [22]. These genetic groups are apparently linked to different habitats [21] where G1 is typically found in dry locations such as fields/meadows, and roadsides, G2 is associated with wet and shady habitats, such as forests and forest margins, ponds and rivers, and G3 seems to be confined to the grass genera *Spartina* and *Distichlis* in saline habitats [21,23,24]. The G3 group has so far been detected in Argentina, Germany, Ireland, the UK, and the USA on coastal salt marshes [22,23,24,25,26]. Furthermore, the different genetic groups reportedly also represent different chemoraces where each group produces different sets of alkaloids. According to Pažoutová *et al.* [21,27], sclerotia of G2 contain equal amounts of ergosine and ergocristine and 5%–15% of ergocryptine. None of the other alkaloids were found. Moreover, G3 sclerotia from *Spartina anglica* included a mixture of ergocristine and ergocryptine. Both G2 and G3 therefore constitute stable chemoraces. However, sclerotia of the G1 group showed no specific alkaloid pattern and contained from one to seven different ergot alkaloids. Noteworthy, G1 contains ergotamines and ergotoxines [21,27]. In the few studies that have been conducted until now, *C. purpurea* genetic groups have showed a remarkable adaptability to the different habitats. Sclerotia produced by G1 confined to dry habitats do not float on water, whereas sclerotia produced by G2 and G3, confined to wet habitats, are able to float for a long time [21,27]. Previous phylogenetic analyses indicate that there is little or no gene flow between the genetic groups. Therefore, it has been hypothesized that habitat-driven evolution is leading to separate species with unique ergot alkaloid chemistry within *C. purpurea* *sensu lato* [22]. Apparently, the host plant has no influence on alkaloid composition in *C. purpurea* sclerotia, but the quantitative amount of the various alkaloids may vary between hosts [21,27]. However, many of the above topics have not been investigated thoroughly, and the geographic coverage of previous studies covers predominantly the Americas and a small part of Europe. No similar studies have been conducted for *C. purpurea* in northern European habitats.

A new study recently proposed the delimitation of cryptic species within *C. purpurea* *sensu lato* [28] based on multi locus phylogenies and gene flow statistics. Here, the G1, G2, and G3 are assigned to the separate species *C. purpurea* *sensu stricto*, *C. humidiphila*, and *C. spartinae*, respectively, whereas a new genetic group from the arundinoid grasses *Molinia* and *Phragmites* has been described as *C. arundinis* sp. nov. However, the new species description did not take alkaloid chemistry into account. The aims for our study were to (1) explore the genetic diversity of *C. purpurea* *sensu lato* in Norway from selected hosts and habitats, (2) characterize the profiles of indole alkaloids (including ergot alkaloids and indole-diterpenes) in relation to genetic groups including recently proposed species, and (3) reveal relationships between genetic groups, chemoraces and ecology.

**Table 1:** Sample overview of *Claviceps purpurea sensu lato* sclerotia used in the study. The number of sampled sclerotia is given in parentheses for each grass host species. Accompanying information regarding origin and habitat is given along with results for identified genetic groups, co-occurrence, and sclerotial floating ability linked to genetic groups.

Year	Location ^a^	Plot	Latitude/longitude	Habitat ^b^	*Host* ^c^ (# sclerotia)	Genetic groups ^d^ (sinking/floating data for sclerotia), & co-occurrence *
2011	**1**	1	58.06502/6.78550	Saline	*Leymus arenarius* (**4**)	G1 (3/1)
		2	58.06642/6.79428	Saline	*Leymus arenarius* (**5**)	G1 (2/0), G2 (1/0), G4 (0/2) *
		3	58.06999/6.72743	Saline	*Calamagrostis epigejos* (**20**)	G2 (20/0)
2012		4	58.07044/6.72735	Saline	*Leymus arenarius* (**15**)	G1 (3/4), G2 (0/3), G4 (0/5) *
2011	**2**	1	58.74609/5.51021	Saline	*Ammophila arenaria* (**13**)	G1 (8/0), G2 (2/3) *
		2	58.88869/5.60333	Saline	*Ammophila arenaria* (**10**)	G1 (10/0)
2011	**3**	1	59.97072/10.74814	Very wet	*Molinia caerulea* (**8**)	G4 (0/8)
		2	59.97174/10.7477	Very wet	*Molinia caerulea* (**20**)	G4 (0/20)
		3	59.97301/10.74723	Very wet	*Molinia caerulea* (**15**)	G4 (0/15)
		4	59.96967/10.74828	Wet	*Phleum pratense* (**5**)	G2 (0/5)
2012		5	59.96972/10.74832	Wet	*Calamagrostis arundinacea* (**15**)	G2 (0/15)
		5	59.96972/10.74832	Wet	*Deschampsia cespitosa* (**11**)	G2 (6/5)
		6	59.96887/10.74753	Wet	*Elymus repens* (**6**)	G1 (6/0)
		7	59.96828/10.74738	Wet	*Phleum pratense* (**10**)	G2 (0/10)
		8	59.96832/10.7481	Wet	*Schedonorus pratensis* (**14**)	G1 (14/0)
2011	**4**	1	59.97219/10.76052	Dry	*Phleum pratense* (**12**)	G2 (0/12)
		2	59.97263/10.76325	Dry	*Phleum pratense* (**16**)	G2 (4/12)
		3	59.97296/10.76408	Dry	*Dactylis glomerata* (**28**)	G1 (5/0), G2 (5/18) *
		3	59.97296/10.76408	Dry	*Elymus repens* (**8**)	G1 (8/0)
		3	59.97296/10.76408	Dry	*Schedonorus pratensis* (**13**)	G1 (13/0)
2012		4	59.9723/10.75967	Dry	*Calamagrostis arundinacea* (**20**)	G2 (0/20)
		5	59.97898/10.75447	Very wet	*Molinia caerulea* (**20**)	G4 (0/20)
2012	**5**	1	58.97152/9.26459	Dry	*Anthoxanthum odoratum* (**15**)	G1 (15/0)
		1	58.97152/9.26459	Dry	*Dactylis glomerata* (**20**)	G1 (20/0)
		1	58.97152/9.26459	Dry	*Festuca rubra* (**20**)	G1 (20/0)
		1	58.97152/9.26459	Dry	*Phalaris arundinacea* (**15**)	G2 (0/15)
		1	58.97152/9.26459	Dry	*Poa pratensis* (**15**)	G1 (15/0)
		2	58.97147/9.26508	Very wet	*Molinia caerulea* (**20**)	G4 (0/20)
		3	58.9682/9.25837	Dry	*Calamagrostis arundinacea* (**29**)	G2 (14/15)
		3	58.9682/9.25837	Dry	*Calamagrostis epigejos* (**34**)	G2 (15/19)
		3	58.9682/9.25837	Dry	*Phleum pratense* (**31**)	G1 (5/0), G2 (4/12) *
2011	**6**	1	59.9838/10.66953	Dry	*Phleum pratense* (**40**)	G2 (20/20)
		2	59.98401/10.66767	Dry	*Phleum pratense* (**36**)	G2 (16/20)
2012		3	59.98268/10.67023	Dry	*Deschampsia cespitosa* (**8**)	G2 (0/8)
		3	59.98268/10.67023	Dry	*Festuca rubra* (**10**)	G2 (0/10)
		4	59.97965/10.66966	Dry	*Calamagrostis arundinacea* (**15**)	G2 (0/15)

**^a^ Location: 1 =** Farsund, Vest-Agder, exposed costal landscape in the Southernmost part of Norway. **2** = Jæren, Rogaland, exposed costal landscape, slightly South-West in Norway, **3** = Korsvoll, Oslo, forest landscape with fresh water lakes and marshes in the South-Eastern part of Norway, **4** = Maridalen, Oslo, including forests and dry open agricultural fields in South-Eastern Norway, **5** = Kurdøla, Telemark, including wet and dry landscapes (forests and natural fields with fresh water lakes and marshes) in Southern Norway (inland), **6** = Tryvann, Oslo, including forests and dry open fields in South-Eastern Norway, ~500 meter above sea level; **^b^** **Habitat: Saline** = salt exposed sandy beach (sand-dune habitat). **Wet** = shady wet forest. **Very wet** = grass roots constantly submerged in water, either in marshes or at lake edges. **Dry** = dry open fields, either natural or edges of agricultural fields; **^c^ Grass host species:** All grass host belong to the subfamily Pooideae with the exception of *Molinia caerulea*, which belongs to the subfamily Arundinoideae; **^d^ Genetic groups** of *Claviceps purpurea* *sensu lato*, according to a recent study [28], are delimited into the following species: G1 = C. purpurea sensu stricto, G2 = C. humidiphila, G3 = C. spartaniae, and G4 (referred to elsewhere as G2a [28]) =C. arundinis sp. nov. Colour codes of the genetic groups in accordance with other figures; * Asterisks indicate co-occurrence of genetic groups on the same host within a single plot within a 2–3 m^2^ area.

## 2. Results

### 2.1. Genetic Groups

In this study 596 full-length sequences of the nuclear rDNA internal transcribed spacer region (ITS), including ITS1, 5.8S and ITS2, were obtained from the collected sclerotia, out of which 15 unique ITS sequences were found. For a subset of 40 samples including all recorded ITS-types, analysis of β-tubulin gene (*tubB*) sequences yielded 11 unique sequences. The *C. purpurea sensu lato* sclerotia analyzed in this study is listed in Table 1. 

**Figure 1:**
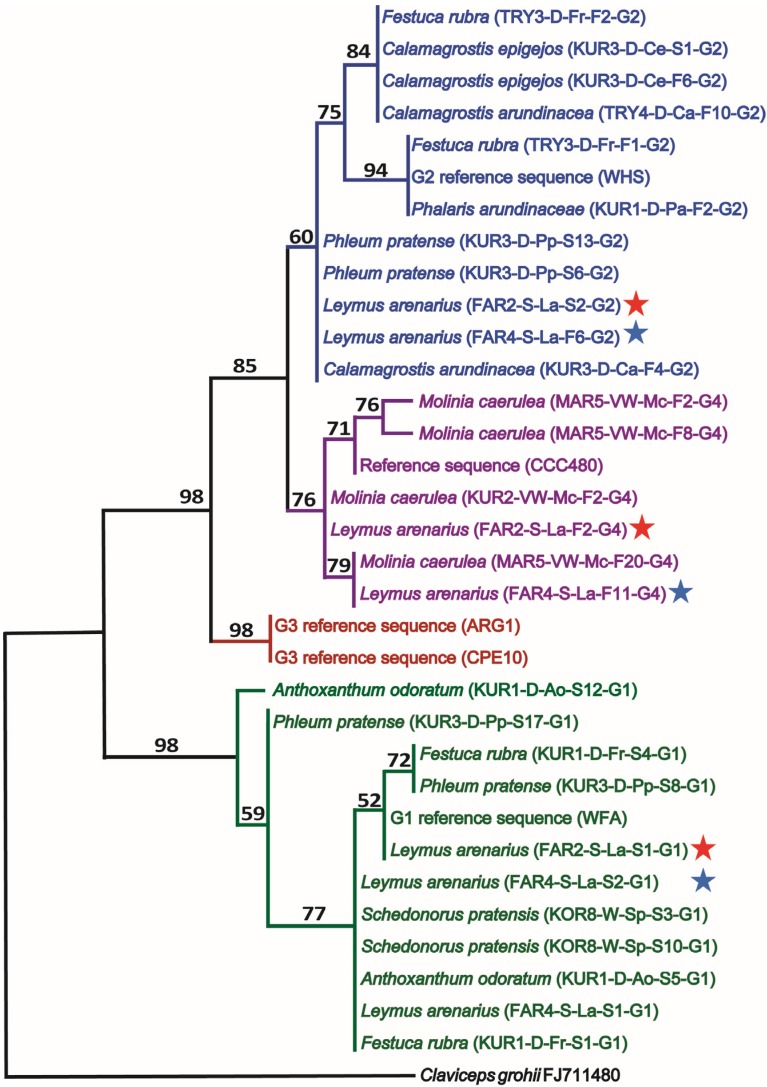
One out of two most parsimonious trees (maximum parsimony, unweighted; length = 59) of the *C. purpurea* *sensu lato* species complex constructed from combined alignments of representative internal transcribed spacer region (ITS) and β-tubulin (*tubB*) DNA sequences. Four major groups (G1 (green), G2 (blue), G3 (red) and G4 (purple)) are identified although the separation of G2 and G4 had less bootstrap support (1000 replicates; number above branches). In the tree, included sequences of this study are denoted with original host of the *Claviceps* sclerotium designated by a sequence ID (in parentheses) encoded as follows: Locality (FAR = Farsund; KOR = Korsvoll; KUR = Kurdøla; MAR = Maridalen; TRY = Tryvann), plot # (1–8) within each location, habitat (D = dry, W = wet, VW = very wet, S = saline), host (Ao = *Anthoxanthum odoratum*, Ca = *Calamagrostis arundinacea*, Ce = *Calamagrostis epigejos*, Fr = *Festuca rubra*, La = *Leymus arenarius*, Mc = *Molinia caerula*, Pa = *Phalaris arundinacea*, Pp = *Phleum pretense*, Sp = *Schedonorus pratensis*), floating ability (F = floating, S = sinking), sclerotia # within the sample, and the genetic group (G1–G4). Stars indicate co-occurrence of three genetic groups within the same plot on the same host over two seasons (2011 = red star, 2012 = blue star). The sequences have been submitted to EMBL/GenBank under the following accession numbers: ITS (LN736206–LN736233), *tubB*: (LN736234–LN736261). Reference sequences from *Claviceps grohii* CBS isolate 124.47 (ITS: AJ133395 *tubB*: FJ711480) were included as outgroups to root the tree. Reference sequences from *C. purpurea sensu lato* retrieved from EMBL include G2 reference WHS (ITS: EU559018, *tubB*: EU558997), CCC480, recently described as *C. arundinis* which grouped with our G4 isolates (ITS: EU344983, *tubB*: JX083420), G3 reference ARG1 (ITS: JX083542, *tubB*: JX083473), G3 reference CPE10 (ITS: EU559007, *tubB*: EU558986) and G1 reference WFA (ITS: EU559017, *tubB*: EU558996).

Phylogenetic analysis of a concatenated ITS/*tubB* matrix revealed four main clades including the previously well-known G1 and G3 groups, and additionally one new genetic group that we are designating G4 (Figure 1). G1 and G3 formed highly supported clades (98% bootstrap support), but the G2 and G4 clades had less support (60% and 76%, respectively). The G2 and G4 clades appeared as sisters in an 85% supported clade. The alkaloid profiles and ecological data (see below) were consistent with the phylogenetic division of G2 and G4. Intra-group genetic variation appeared in all four main groups.

**Figure 2:**
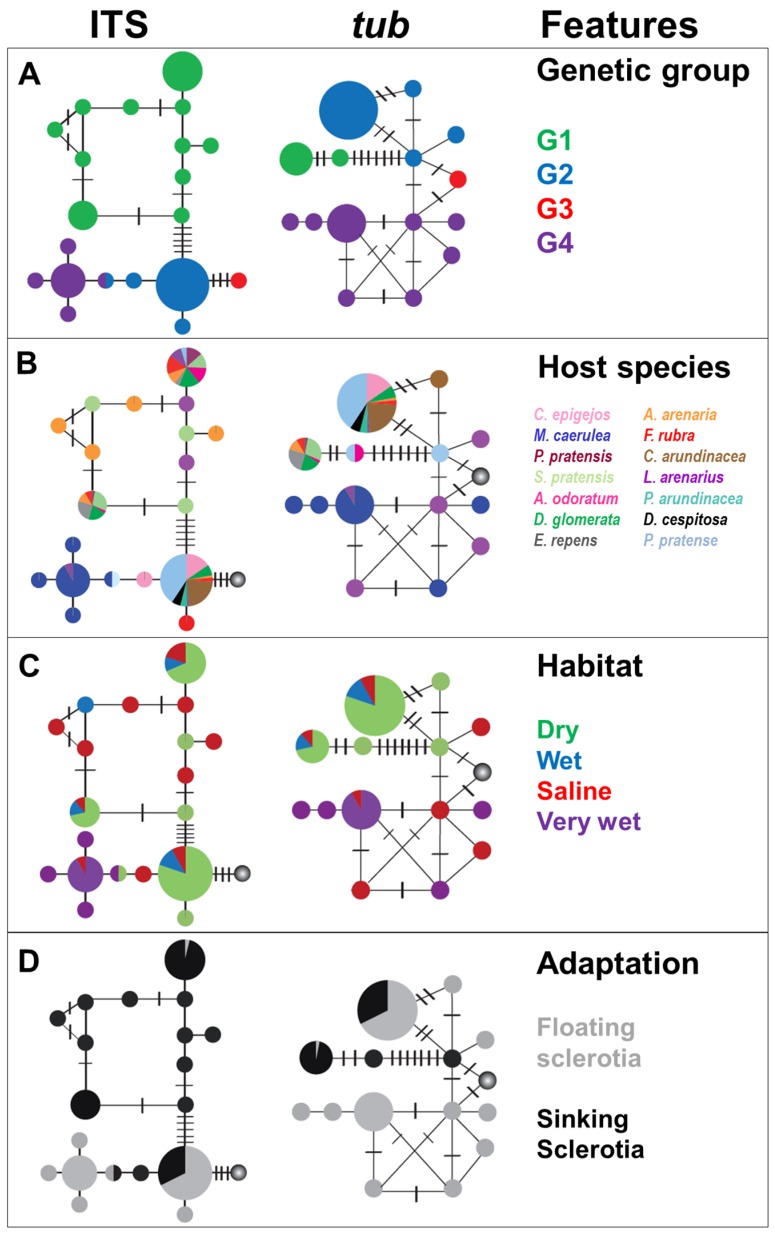
Haplotype networks of unique ITS (left) and *tubB* (right) sequences of the *C. purpurea sensu lato*. Each circle represents one haplotype and there is one mutational step between each circle and short crossing line (representing hypothetical haplotypes). Circle size reflects the haplotype abundance ranging from approximately *N* = 1 to *N* = 350. In the different panels, the following information (features) is superimposed: (**A**) genetic groups inferred from the phylogenetic analysis (cf. Figure 1), (**B**) host plant, (**C**) habitat, and (**D**) adaptation in terms of floating ability of the sclerotia. The circle representing G3 is shaded with a grey gradient in panels B–D to indicate lack of data.

Haplotype networks were constructed separately for the ITS and *tubB* datasets (Figure 2). The networks, depicting the distribution of the genetic groups G1-G4 (Figure 2A), was consistent with the phylogenetic results in that the G1 group formed a separate assemblage that was separated from the G2 group by 6–8 mutational steps. Fewer mutational steps separated the G2 and the G4 group, and these had one haplotype in common in the ITS network, but were well-separated in the *tubB* network. The G3 group appeared to be more closely related to G2 than to the others, and the G2 and G3 clades were separated by four changes in ITS and two changes in *tubB*. Within each genetic group several haplotypes were found in both networks and also some reticulate patterns, possibly reflecting intra-group recombination (intra-locus). It is noteworthy that no reticulate patterns were seen across groups, indicating that the different groups were genetically isolated from one another. The ecological characteristics superimposed onto the networks are addressed further in section 2.3.

**Figure 3:**
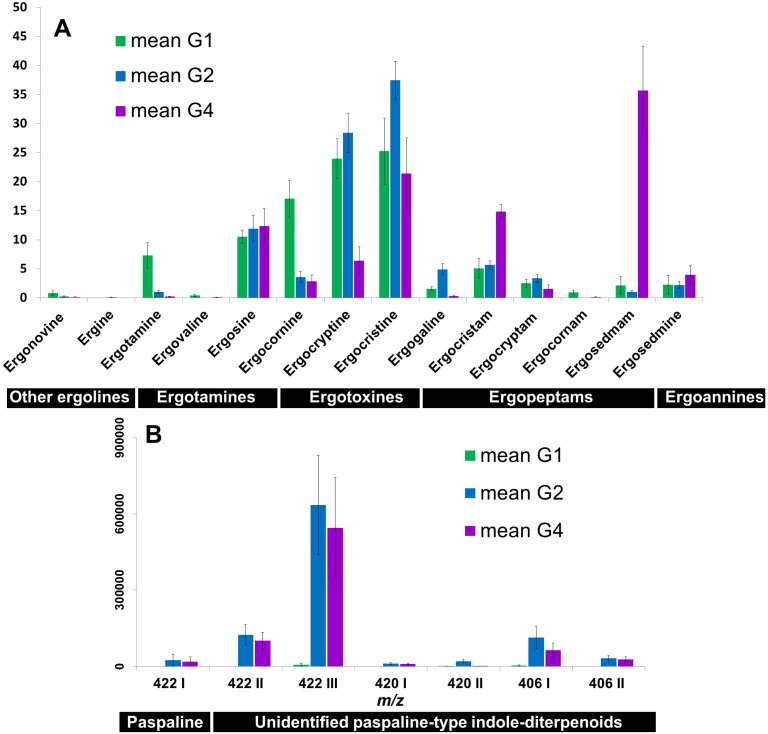
Histograms showing relative concentrations of ergot alkaloids (sum of 8-*R* and 8-*S* epimers) (**A**) and indole-diterpenes (**B**) in the three examined genetic groups of *C. purpurea sensu lato* of this study (G1, G2 and G4). Chemical structures of most *C. purpurea sensu lato* indole-diterpenes are not yet determined, and designations are thus according to the *m/z* ratio of their protonated molecular ions and their elution order. For an overview over chemical structures see Figure S1 and [29,30].

### 2.2. Indole Alkaloid Profiles

The indole alkaloid profiles in the *C. purpurea sensu lato* sclerotia were characteristic for each genetic group (Figure 3). The chromatograms from the HPLC-PDA-MS analyses demonstrated that the alkaloid profile in sclerotia of the genetic group G2 was largely dominated by ergocristine and ergocryptine. Similarly, sclerotia from the G1 group contained mostly these two ergotoxines. However, the sclerotia of the G1 group contained considerably higher relative concentrations of other ergopeptines such as ergotamine and ergocornine. The relative concentrations of ergopeptams (ergocristam and ergosedmam) in G4 sclerotia were considerably higher than in the other two groups (Figure 3A). Specifically, the G4 sclerotia contained ergosedmam as the principal ergot alkaloid, which was tentatively identified earlier as the lactam congener of ergosedmine [6]. Ergopeptams are the lactam congeners of ergopeptines (Figure S1). In ergopeptine biosynthesis, the last step is the spontaneous cyclolization of the amino acid next to the D-lysergyl moiety after hydroxylation of the α-carbon by the enzyme EasH [31]. It has recently been shown that EasH can cyclolize ergotamines, ergoxines and ergotoxines [31]. Before cyclolization, however, the L-proline in the tripeptide can partly isomerize to D-proline. The resulting D-prolyl stereoisomer is then no longer cyclolized by EasH, which is the reason for the natural occurrence of the ergopeptams. G4 sclerotia differed further from G2 sclerotia by a relatively smaller content of ergocryptine (Figure 3A). The G1 sclerotia contained only traces of indole-diterpenes, whereas indole-diterpene production in G2 and G4 was pronounced and the profiles were similar to each other (Figure 3B). The major indole-diterpene was a compound apparently affording protonated molecular ions at *m/z* 422 [20]. Furthermore, the HPLC-MS chromatograms showed the presence of two other *m/z* 422 compounds, of which one corresponded to paspaline (verified with authentic standard) [20]. The remaining *m/z* 406 and *m/z* 420 indole-diterpene compounds are yet unidentified congeners with structures similar to paspaline [20] (Figure 3B).

**Figure 4:**
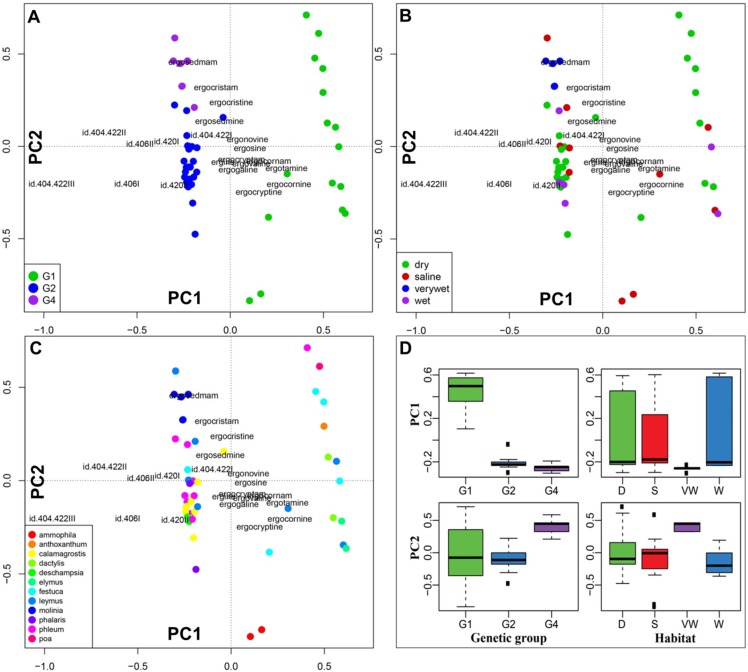
Principal component analysis (PCA) of fourth root transformed indole-alkaloid profiles shown as biplots with sample scores as dots and indole-alkaloid loadings as text labels. The sample score colours are coded according to (**A**) genetic group, (**B**) habitat, and (**C**) host plant genus. Box-plots (**D**) compare the first and second principal components for scoring of the genetic groups and habitat (D = dry, S = saline, VW = very wet and W = wet) illustrating that the first PC divides G1 from G2/G4 and the second PC separates G2 from G4. Note: *Schedonorus pratensis* (= *Lolium pratense =* *Festuca pratensis*) was treated as *Festuca.*

Principal Component Analysis (PCA), based on the alkaloid profiles (Figure 4) clustered the genetic groups, reflecting a high consistency between the genetic and chemical data. The PC1 axis divided G1 from G2/G4, and PC2 separated G2 from G4 (Figure 4A, D). All three candidate explanatory variables (genetic group, genus, habitat) explained a significant fraction of the Euclidean distance between alkaloid profiles, according to multivariate permutation tests (PERMANOVA, Table 2). Genetic group and host genus explained more of the total variance than habitat. The lower number of degrees of freedom among genetic groups (*Df* = 2) compared to host genera (*Df* = 11), makes genetic group the most robust and parsimonious predictor of *Claviceps* alkaloid composition (c.f. the F values; Table 2). The explanatory variables are confounded, e.g., G4 occurs almost only on *Molinia* that exclusively was found in very wet habitats. This lack of independence precludes the use of interaction terms on the models, even though such would have been potentially useful for untangling genetic and environmental effects on alkaloid profiles. 

**Table 2:** Multivariate permutation tests (PERMANOVA) on Euclidean distances between indole-alkaloid profiles. The used explanatory variables were genetic group, host genus, or habitat. Columns represent degrees of freedom in the explanatory variable (*Df*), un-permuted *F* ratios of the model (F.model), fraction of total variance explained by the model (R2), and a permutation-based significance probability of the model *F* ratio (Pr(>*F*); 999).

Explanatory variable	*Df*	F.Model	R2	Pr(>*F*)
Genetic group	2	22.369	0.49854	0.001
Host genus	11	2.8885	0.46882	0.001
Habitat	3	2.4141	0.14134	0.013

### 2.3. Ecological Characteristics 

The haplotype networks illustrated some differences in host affinity between the three genetic groups (Figure 2B). G1 was associated with *Poa pratensis*, *Schedonorus pratensis*, *Anthoxanthum odoratum*, *Dactylis glomerata*, *Elymus repens*, *Ammophila arenaria*, *Festuca rubra*, *Leymus arenarius* and *Phleum pratense*, whereas G2 was associated with *Calamagrostis epigejos*, *D. glomerata*, *A. arenaria*, *F. rubra*, *Calamagrostis arundinacea*, *L. arenarius*, *Phalaris arundinacea*, *Deschampsia cespitosa* and *P. pratense.* Hence, the plant species, *P. pratensis*, *S. pratensis*, *A. odoratum* and *E. repens* hosted exclusively the genetic group G1, whereas the plant species *C. arundinacea*, *C. epigejos*, *D. cespitosa* and *P. arundinacea* hosted exclusively the genetic group G2. On the other hand, G4 was only found on two host plant species, predominantly and consistently on *Molinia caerulea* in very wet habitats, but also on *L. arenarius* in saline sand-dune habitats that simultaneously hosted both G1 and G2 (Table 1).

On several occasions the sampled sclerotia from one specific location and within a plot (= an area of maximum 2–3 m^2^) belonged to multiple genetic groups (Table 1). It is noteworthy that, during both sampling seasons of this study (2011 and 2012), the three genetic groups G1, G2 and G4 were present on the same host plant, *L. arenarius*, in a single plot within the saline sand-dune location at Farsund in South Norway, demonstrating sympatry in both space and time. 

The PCA plot did not reveal any clear connection between the host plant and the alkaloid profiles (Figure 4C). Although genus explained almost 47% of the total variance and was found to be a significant explanatory variable for the Euclidean distance between alkaloid profiles (Table 2), the *F*-value was low due to the high number of degrees of freedom for hosts (*Df* = 11). In a few cases, the plot showed a tendency of clustering alkaloids by some of the host plants. The alkaloid profiles from *Calamagrostis* spp. (*C. arundinacea* and *C. epigejos)* clustered together, as did the five alkaloid profiles from *Molinia caerulea*. 

**Figure 5:**
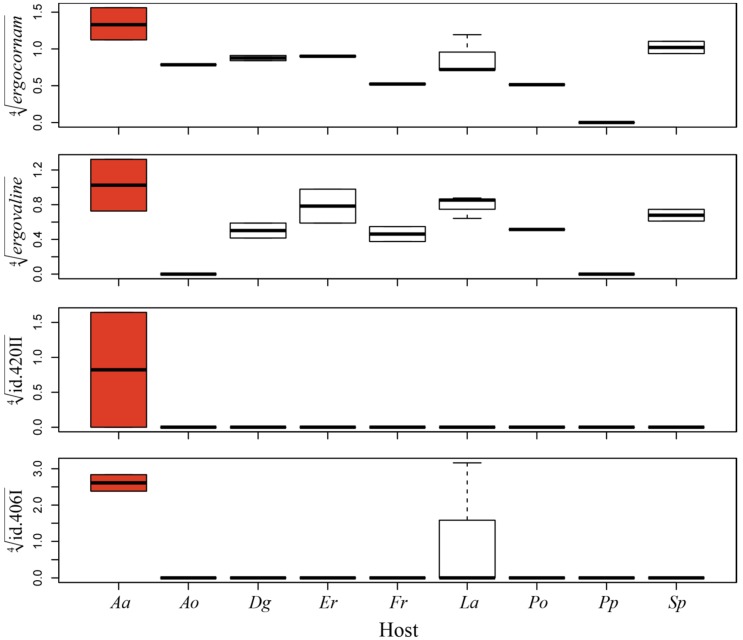
Unique indole-alkaloid composition in G1 sclerotia of *Ammophila arenaria*. The box-plots (fourth root transformed relative indole-alkaloid concentrations) show the four indole alkaloids where G1 sclerotia of *A. arenaria* produce significantly (red boxes) higher quantities compared to G1 sclerotia of host plant species. Host abbreviations: *Aa = Ammophila arenaria Ao = Anthoxanthum odoratum*, *Dg = Dactylis glomerata*, *Er = Elymus repens*, *Fr = Festuca rubra*, *La = Leymus arenarius*, *Po = Poa pratensis*, *Pp = Phleum pretense*, *Sp = Schedonorus pratensis*.

Although genetic group was the most robust and parsimonious predictor of *Claviceps* alkaloid composition, exceptions were observed for one host genus. Ergovaline was present in high relative quantities in G1 sclerotia found on *A. arenaria* in a saline habitat, but only present in trace amounts in G1 sclerotia from other hosts (*t* = 3.26, *p* = 0.0057). The same pattern was found for ergocornam (*t* = 4.265, *p* = 0.0008). Thus, the host plant *A. arenaria* seemed to significantly influence the production of certain ergot alkaloids. This was also the case for some of the indole-diterpenes (*m/z* 420 II [(*t* = 3.5, *p* = 0.0035] and *m/z* 406 I [(*t* = 2.065, *p* = 0.058)]), which were practically absent in the extracts from most G1 sclerotia other than those from *A. arenaria* (Figure 5). Also, for three other ergot alkaloids, we observed substantial (although not significant) differences in the composition of the alkaloid mixture in G1 sclerotia from different hosts. Ergonovine (*t* = −0.646, *p* = 0.53) was produced in G1 sclerotia from *D. glomerata*, *F. rubra* and *A. odoratum*, and present only in trace amounts in G1 sclerotia from all other hosts. Ergocornine (*t* = 0.639, *p* = 0.53) was largely present in all G1 sclerotia with lesser relative amounts in those that originated from *F. rubra*, *P. pratensis*, *P. pratense* and *A. odoratum*. Ergotamine was also detected in all G1 sclerotia, though in lower relative amounts (*t* = 1.626, *p* = 0.13) than ergocornine, and mostly in sclerotia from *A. arenaria* and *L. arenarius* (Figure S2).

**Figure 6:**
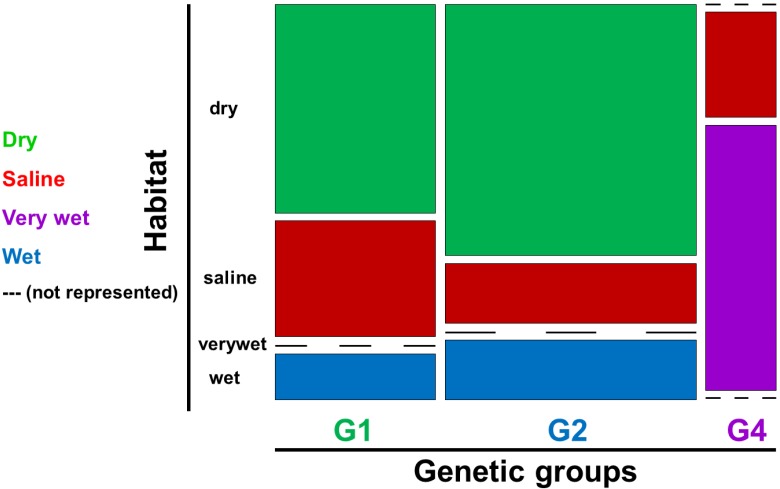
Mosaic plot of habitat distribution across the genetic groups, where G1 and G2 both were found in dry, wet and saline habitats in Norway. In contrast, the genetic group G4 was the only group with high prevalence in very wet habitats. G4 was infrequently found also in saline habitats.

We did not find any clear relationship between habitat and genetic groups (Figure 2C, Figure 4B,D). In the haplotype networks, the different habitat types were distributed in a rather random manner, reflecting little of the previously described preference of G1 for dry habitats, and of G2 for wet habitats. Although many of the sclerotia collected in dry habitats represented G1 sclerotia, a large portion of G1 sclerotia also originated from saline and wet habitats. Similarly, the G2 sclerotia were also in part collected in dry and saline habitats (Figure 2C). The genetic group G4 was more homogenous in this respect, occurring mostly in very wet habitats and occasionally in saline environments. The habitat affiliations of the genetic groups are further visualized in a mosaic plot, which more clearly shows the similarity in the habitat distribution of G1 and G2 (Figure 6). The rather low preference of the genetic groups for a specific habitat was further reflected in the general absence of a connection between habitat type and alkaloid profile in the PCA plot. The exception was again the very wet habitat, which was associated with the genetic group G4 (Figure 4B,D). 

There was a clear correspondence between the ability to float and genetic affiliation, where sclerotia from G1 generally sank (97%), and the sclerotia from G4 consistently floated (100%; Table 1, Figure 2D). Surprisingly, sclerotia from the G2 group were more variable in their ability to float. Most G2 sclerotia showed the ability to float (66.95%), but a large portion also sank (33.05%). The ability of the G2 sclerotia to either float or sink was significantly related to host (Fisher’s exact test, *p* = 0.0025). However, the sample from various host genera was very unbalanced, with large variations in the number of sclerotia. Thus, if only the three most abundant genera within G2 were included in the test (*Phleum*, *Calamagrostis*, *Dechampsia*), no significant relationship was found (Fisher’s exact test, *p* = 0.40). The relative proportion of sinking G2 sclerotia in dry habitats (35.5%, including sclerotia from the saline habitat that in our study represented very dry salt exposed sand dunes) was significant higher (Fisher’s exact test, *p* = 0.0076) than in wet habitats (14.6%). This sample was less unbalanced, although the collection of G2 sclerotia from dry habitats (including saline) was eight-fold larger than for G2 sclerotia from wet habitats. 

## 3. Discussion

### 3.1. Genetic Groups and Speciation

*Claviceps purpurea* was until recently considered a single wide-ranging and morphologically variable species, including genetic lineages with specific habitat preferences, ecological adaptations and alkaloid chemistry [21,22,27]. Very recently, Pažoutová *et al.* [28] have proposed a formal delimitation of these lineages, or cryptic species, into four biological species based on multi-locus phylogenies and gene flow statistics in adition to ecological and morphological characteristics. Our study supports and complements the proposed delimitation both genetically and with new indole-alkaloid data, but also adds some considerations regarding host spectra, habitat preferences and adaptations. The recently published species names are not yet widely known. We will therefore refer species name and the corresponding genetic group together; *i.e.*, *C. purpurea* *sensu stricto* (G1), *C. humidiphila* (G2), *C. spartaniae* (G3) and *C. arundinis* (G4), and for simplicity sometimes only refer the genetic groups alone. 

Our multi-locus phylogeny together with the haplotype networks supported the proposed species delimitation [28] including *C. arundinis* sp. nov. (G4) that has not been reported in Norway before. One conflicting result was, however, that G4 and G2 share one haplotype. On the other hand, some shared genetic variation is expected in recently diverged lineages due to e.g. incomplete lineage sorting. In Pažoutová *et al.* [28], the multi-locus phylogenetic analysis with concatenated data (GCPSR) did not entirely resolve G2 and G4 (in that paper denoted G2a), but population genetic analysis confirmed the four recognized species with unique and fixed polymorphisms (9.3% fixed nucleotide positions differentiating G2 and G4). They concluded that *C. arundinis* (G4), still sharing polymorphism in rDNA with *C. humidiphila*, is an incipient species with an estimated divergence from *C. humidiphila* (G2) of ~1.17 Ma. In our study, the alkaloid profiles, and also the ecological data in part, supported G4 as a recently evolved, but well-defined species characterised by its preference for *Molinia caerulea* and production of ergosedmam (see below).

### 3.2. Alkaloid Profiles and Speciation

The PCA and PERMANOVA analyses indicated that alkaloid profiles are linked to the genetic groups within *C. purpurea sensu lato*, and identified the affiliation to a specific genetic group as the most robust and parsimonious predictor of *Claviceps* alkaloid composition. Thus, the alkaloid gene clusters would probably be well suited for studying evolution and diversification of alkaloid chemistry and speciation within the species complex. Comparative genomics analysis of the Clavicipitaceae has revealed a strong tendency for alkaloid loci to have conserved cores, specifying the skeleton structures and peripheral genes that determine chemical variations, which in turn can affect their pharmacological specificities [4]. Much of the variation determining ergot alkaloid diversity is in genes for the non-ribosomal peptide synthetase (NRPS) subunits in lysergyl peptide synthetase enzyme complexes (reviewed in [29,30]). These complexes can include LPS2 with LPS3 subunits, specifying ergonovine, or LPS2 with LPS1 subunits specifying ergopeptide lactams, which are converted to ergopeptines by action of the EasH oxygenase. A study comparing the cluster sequences of an ergotamine producer (strain P1) with an ergocristine producer (strain ECC93 [32]) of *C. purpurea* found significant variation in the NRPS modules, suggesting that the recent and ongoing diversification and evolution of “chemical races” of *C. purpurea* *sensu lato* is determined by evolution of NRPS module specificity [4]. For example, it has been demonstrated that two lpsA genes for LPS1 subunits specify different ergopeptide lactams, where lpsA1 specifies ergotamam (for ergotamine) and lpsA2 specifies ergocryptam (for ergocryptine) [33].

In agreement with Pažoutová *et al.* [21] we found that ergocristine, ergocryptine and ergosine were the major ergopeptines in sclerotia of *C. humidiphila* (G2). However, the three analogues were consistently present in the order ergocristine > ergocryptine > ergosine, and not ergocristine > ergosine > ergocryptine as previously reported [21]. According to earlier reports, sclerotia of *C. purpurea* *sensu stricto* (G1) contain mixtures of ergotamines and ergotoxines. This is to some degree in accordance with our results, *i.e.*, the relative occurrences of ergotamines and the ergotoxine ergocornine were significantly higher in G1 compared to G2 sclerotia. However, although the relative amount of ergocristine and ergocryptine was highest in G2, these were still the dominant ergopeptines in G1 sclerotia. The ergot alkaloid profile of *C. arundinis* (G4) sclerotia differed strongly from G1 and G2 sclerotia by the dominance of ergopeptide lactams, and also that the major ergopeptine in G4 was ergocristine. This is unexpected since G4 sclerotia apparently contained the lactam congener of ergosedmine (“ergosedmam”) as the principal ergot alkaloid. It could therefore be expected that the major ergopeptine in these sclerotia is ergosedmine. However, G4 sclerotia were found to contain only minor amounts of this analogue. A possible explanation for this observation could be that cyclol formation with the relatively bulky *sec*-butyl side chain of isoleucine in amino acid position 1 [29] is not favoured because of steric hindrance. 

The G2 (*C. humidiphila*) and G3 (*C. spartaniae*) have previously been defined as stable chemoraces that produce only ergosine, ergocristine and smaller amounts of ergocryptine (G2), ergocristine and ergocryptine (G3) [21]. However, we demonstrated that G2 sclerotia contained several other ergopeptines, although in lower relative concentrations than in G1 sclerotia. This might in part be explained with lower detection limits of our LC-MS based approach *vs.* earlier methods that were usually based on HPLC separation and fluorescence or UV-absorbance detection. Though fluorescence detection commonly provides especially low detection limits, mass spectrometric detectors are more specific and thus enable detection of low-abundance compounds with higher confidence. 

The indole-diterpenes, well known from other *Claviceps* species and from the sister genus *Epichloë* (including *Neotyphodium* spp.) [16,17,34,35], have not received any attention in *C. purpurea sensu lato*, until recently when *C. humidiphila* (G2) sclerotia were reported to contain indole-diterpenes tentatively identified as paspaline analogues [20]. The present study demonstrates that both *C. humidiphila* (G2) and *C. arundinis* (G4) produce extensive amounts of indole-diterpenes, whereas these are merely absent in *C. purpurea* *sensu stricto* (G1) with the exception of G1 sclerotia on *A. arenaria* that produced small amounts of two unidentified paspaline-like indole-diterpenes. For indole-diterpene producing strains of Clavicipitaceous fungi, the indole-diterpene gene clusters (*IDT*) have conserved cores containing the four genes for synthesis of paspaline (*idt/ltmG*, *idtM*, *idtB*, and *idtC*) that is the first fully cyclized intermediate in the indole-diterpene biosynthesis [4]. Presence or absence and sequence variation of the other genes of the *IDT* gene cluster determine the particular forms of indole-diterpenes produced. For example, two more peripheral genes (*ltmE* and *ltmJ*) are present in the lolitrem producing endophyte *Epichloë festucae* [4]. Since we found paspaline-like compounds in sclerotia of both *C. humidiphila* (G2) and *C. arundinis* (G4), we postulate that functional genes for synthesis of paspaline are present in these species, and that key genes (such as *idtG*) are absent in *C. purpurea sensu stricto* (G1) as predicted for the genome sequenced *C. purpurea* strain 20.1 [4], which most likely represents a G1 isolate. Thus, the *IDT* cluster remains to be explored for *C. humidiphila* (G2), *C. spartaniae* (G3) and *C. arundinis* (G4), and it would be of interest also to screen for *C. purpurea sensu stricto* on *A. arenaria* since these G1-sclerotia contained minor but detectable amounts of indole-diterpenes. Biosynthetically more complex indole-diterpenes—e.g., paspalitrems—were not detected in any of the *C. purpurea sensu lato* lineages*.*

Pažoutová *et al.* [21,27] hypothesized that the production of a specific ergot alkaloid are not influenced by the host plant species, whereas the relative ratios of the various alkaloids might be affected by the host. Our results support this hypothesis, since there was no clear association between the overall alkaloid profiles and the host plant species. However, exceptions were observed. The G1 sclerotia of the saline-adapted grass *A. arenaria* differed significantly from G1 sclerotia from all other hosts by containing higher amounts of ergovaline, ergocornam and two unidentified indole-diterpenes. The indole alkaloid composition and quantity for this specific case may depend both on fungal genotype and on the host plant. 

### 3.3 Ecology and Speciation

Pažoutová *et al.* [21,27] suggested that the genetic groups G1–G3 were host specialised to some degree. In their studies it was found that the species *A. arenaria*, *Dactylis* sp., *F. rubra* and *Phleum* sp*.* were common hosts both for G1 and G2, while species of *Calamagrostis* and *Phalaris* hosted only G2. Moreover, the G3 genetic group has been recorded only from the genera *Spartina* and *Distichlis* [21,23,24]. A thorough host range has been given for the new *C. purpurea sensu lato* species descriptions [28]. In short, the typical host of *C. purpurea sensu stricto* (G1) is *Secale cereale* and land grasses of the subfamily Pooideae growing in fields and open meadows or grasslands in temperate regions. The typical host of *C.* *humidiphila* (G2) is grasses from the subfamily Pooideae growing on meadows, wet meadows, river banks, and in forests. Many grass species are shared for G1 and G2, for example *Alopecurus*, *Phleum*, *Dactylis*, and *Poa* spp. [28]. In our study, co-occurrence of G1 and G2 was also observed on the salt tolerant species *A. arenaria* and *L. arenarius* inhabiting very dry sand-dune habitats of the Norwegian coastline. *Calamagrostis* spp., *Deschampsia caespitose* and *Phalaris arundinacea* were found to be infected exclusively by G2 [21,27,28], which is in accordance with our results. The absence of *C. spartaniae* (G3) from our study is not surprising since the G3 hosts are missing in Northern Europe [26,28], and the saline habitats visited during this study are far less extreme in terms of salinity than the salt marshes where G3 has been reported to occur [21,23,24,25,28]. The new species, *C. arundinis* (G4), was, in our study, predominantly associated with *Molinia caerulea*, which is often found as the sole grass species in very wet habitats in Norway. This is in concordance with the species description referring to a narrow host range on the Arundinoid grass hosts *Phragmites* and *Molinia* [28]. However, our records of *C. arundinis* (G4) on *L. arenarius* corroborates the single report of *C. arundinis* from *Agrostis* (Pooideae) [28] in broadening the known host range to include some pooid grasses. 

Correlations between genetic affiliation and habitat preference [21,27] have been reported as typical species characteristics [28] linking *C. purpurea sensu stricto* (G1) to dry open fields, *C.* *humidiphila* (G2) to wet and shady forests and *C. spartaniae* (G3) to saline coastal marshes. However, our results do not provide any clear link between genetic affiliation and habitat preference. We recorded G1 and G2 in wet, dry and saline habitats, and did not find statistic support for habitat segregation. This may partly be due to the spatial structure and high heterogeneity of the habitats in Norway. In some of the investigated habitats, there were both dry and wet areas nearby thus allowing colonization and success of species with both preferences. Further, our definition of “dry” might not be equivalent and comparable to the very dry habitats in the referred literature. Intriguingly, G1, G2 and G4 consistently occurred on *L. arenarius* growing on salt exposed, very dry sand dunes along the Norwegian coastline where this grass helps to stabilize the dunes. The observed co-occurrence of these three *Claviceps* species on *L. arenarius* in both sampling seasons within a single plot strongly supports complete speciation, and our hypothesis that intersterility barriers have evolved. Otherwise, genetic recombination would be expected, for which there was no evidence. The co-occurrences also indicated that interspecific competition might occur. 

The different abilities of the sclerotia to float or sink in water are believed to be caused by differences in the intercellular spaces [36]. In concordance with other studies [21,27,28], we observed a clear correlation between the ability of a sclerotium to float and phylogenetic affiliation of the species. The floating ability of G4 is clearly an adaptation to water-dispersal, whereas this adaptation is not necessary in G1, which mainly is found in drier habitats and grass lands [28]. However, in contrast to the previous reports [21,27,28], we did not find that G1 sclerotia always sank in freshwater or that G2 sclerotia always floated. Although G1 predominantly followed the previously reported patterns, 3% of the G1 sclerotia floated on water (all collected from *L. areanrius*) and 33% of the G2 sclerotia sank (including sclerotia from *A. arenaria*, *P. pratense*, *Calamagrostis arundinacea*, *C. epigejos* and *D. cespitosa*). Exceptions from the sinking *vs.* floating pattern have previously been reported only for floating G1 sclerotia collected from *Glyceria* and *Melica* in wet habitats [28]. The relative large portion of sinking G2 sclerotia may be explained by an adaptation to mixed habitats in Norway (and probably elsewhere) where both abilities can be beneficial. Despite the somewhat unbalanced sample, our data indicated that dry habitats including the saline sand dunes hosted a larger proportion of sinking G2 sclerotia than did the wet habitats. Thus, the current perception of *C. humidiphila* (G2) as a species characterized by floating sclerotia and clear preference for wet and shady habitats [28] should be slightly modified. At least in Norway, G2 is seemingly abundant regardless of habitat type, possibly with a hedging strategy for sclerotial adaptation or alternatively genetic variation for sclerotium type. 

Host specialisation is often seen in biotrophic parasitic fungi such as rusts and smuts, possibly due to the evolutionary arm-race between host and parasite [37]. We may therefore speculate that *C. spartaniae* (G3) and *C. arundinis* (G4) have a more antagonistic nature than *C. purpurea sensu stricto* (G1) and *C. humidiphila* (G2), whereas the latter species may have more of a beneficial role for their host plants, as previously suggested for *C. purpurea sensu lato* [38,39]. Other explanations for host specialisation could involve ecological adaptation and segregation in space, time or both. The occurrence of G3 in salt marsh habitats where the species seems to have co-evolved with the salt-adapted grass genera *Spartina* and *Distichlis* suggests that spatial separation and ecological segregation has been a speciation mechanism for this group. According to (very approximate) molecular dating, the clade of wet-adapted species (G2, G3 and G4) diverged from the lineage to *C. purpurea sensu stricto* (G1) ~7.83 Ma BP in the Late Miocene [28], whereas *C. spartinae* diverged from the G2–G4 group ~2.9 Ma BP, and *C. humidiphila* and *C. arundinis* diverged ~1.17 Ma BP [28]. We found *Molinia caerulea*, the most dominant host for *C. arundinis* (G4) in our study, consistently as the sole grass species in very wet habitats such as waterfront of lakes and rivers or boreal forest swamps and marshes where roots of *M. caerulea* are usually totally submerged in water. This habitat is more extreme than the generally “wet and shady” habitats reportedly preferred by G2 [21,27]. Furthermore, *M. caerulea* sets flowers later than the typical G1 and G2 grass hosts [40]. Hence, temporal separation could also be a contributing factor for the speciation within the G2, G3 and G4 clade [28]. However, the other common grass species from which *C. arundinis* (G4) has been reported frequently is *Phragmites australis* [28]. This cosmopolitan wetland grass is one of the most widely spread plant species in the temperate region of the world. It occurs in both freshwater and saltwater marshes and has evolved ecotypes, some with invasive tendencies, and with resistance to different environmental stressors [41]. Hence, *C. arundinis* (G4) is probably adapted to very wet habitats regardless of salinity. Our record of *C. arundinis* (G4) on *L. arenarius* growing in very dry salt-exposed sand-dunes is therefore interesting, difficult to interpret, and extends the habitat range beyond the very wet habitats. One speculation out of many includes the possibility that *L. arenarius* was an evolutionary precursor host for *C. arundinis* (G4) before G4 established in very wet habitats on *P. australis* (saline and freshwater marshes) and *M. caerulea* (freshwater marshes and boreal forest swamps).

## 4. Materials and Methods

### 4.1. Sampling and Preparation of Sclerotia

*Claviceps purpurea sensu lato* sclerotia were collected from 14 common grass hosts during the Autumn 2011 and 2012, at six locations in Southern Norway, representing four different habitats with various levels of wetness and salinity (Table 1). If possible, the same grass species were targeted between habitats, but different host species were commonly represented in the different habitats. From each plot (2–3 m^2^) 5–15 infected grass individuals per host species were collected (maximum 50 per grass species within a plot). The sclerotia were cleaned of debris, dissected and stored in petri dishes at room temperature. However, in many cases few sclerotia could be recovered from a host plant within a plot, occasionally resulting in small sample sizes from specific hosts (Table 1). In total, 596 sclerotia were included in the analyses. The sclerotia were first submitted to a floating test [21] where the sclerotia were placed in purified H_2_O for 48 hours to reveal their abilities to float or sink. The sclerotia were then air dried and cut in half. One piece of a sclerotium was subjected to DNA analyses, and the other was stored for later indole-alkaloid analyses.

### 4.2. Molecular Analyses

DNA was extracted from one half piece of each sclerotium, using a CTAB extraction protocol [42], including the modifications described by Vrålstad *et al.* [43], and with some minor modifications for this study as follows: 100 μL CTAB buffer (20 g/L, Calbiochem, Darmstadt, Germany; 1.4 M NaCl, 0.1 M Tris-HCl, 20 mM Na_2_-EDTA) was added to each of the Eppendorf tubes containing sclerotium-halves and heated to 95 °C for 5 min, then crushed manually with a pistil (cleaned with household bleach and EtOH, and autoclaved), and then another 600 μL CTAB was added. Samples were placed into a –70 °C freezer for at least 10 min, then removed and heated to 65 °C (5–10 min, until thawed), and centrifuged for 10–15 s. During the DNA extraction two controls were established; extraction blank control (EBK), to check for any carry-over contamination, and extraction environment control (EEK) to check for contamination from the laboratory environment. EBK consisted of a DNA-free tube that followed all steps of the extraction protocol, and EEK consisted of a tube with 200 μL pure H_2_O that was left open during all work with the samples in the laboratory. EBK and EEK were taken through all subsequent steps for PCR analysis. 

### 4.3. PCR and Sequencing

For amplification of the ITS region, primers ITS1 and ITS4 [44] were used. The puReTaq Ready-To-Go PCR Beads kit (Amersham Biosciences, Buckinghamshire, UK) were used for PCR according to the producer’s directions. To each tube 23 μL mastermix was added, consisting of 17 μL pure H_2_O and 3 μL of each primer in a final concentration of 1.7 µM, and 2 μL DNA template. Negative PCR controls were run to control the purity of the H_2_O and reagents used. PCR was performed on a DNA Engine Dyad^®^ Peltier Thermal Cycler (PTC-0220, MJ Research, Waltham, MA, USA); the mixture was first denaturation at 95 °C for 5 min, followed by 35 cycles with a denaturing step at 95 °C (30 s), then annealing at 56 °C (20 s), and synthesis at 72 °C (30 s), and lastly a final elongation at 72 °C (5 min). 

From a smaller subset of the samples representing unique ITS sequences and the three recorded ITS genetic groups, a part of the β-tubulin (*tubB*) gene was sequenced. The *tubB* fragment was amplified with the primers Bt-3NeoF and Bt-3NeoR [45] using the PCR kit as described above. The setup for *tubB* was as follows, a pre-denaturation at 94 °C (4 min), and then 35 cycles of denaturation at 94 °C (30 s), followed by an annealing step at 66 °C (30 s), and synthesis at 72 °C (1 min), and lastly a final elongation at 72 °C (5 min). The PCR products were purified from 5 µL of the mixture with 2 μL Exosap-IT (Amersham Biosciences), according to manufacturer’s instructions. The purified ITS PCR products were diluted 10 times with pure H_2_O, and finally, 9 μL diluted PCR product and 1 μL of each respective primer was added to a new set of plates. The PCR products were sequenced in both directions using ABI BigDye Terminator sequence buffer, v3.1 Cycle sequencing kit and an ABI PRISM^®^ 3730 Genetic Analyzer or an ABI PRISM^®^ 3100 – Avant Genetic Analyzer (Applied Biosystems, Life Technologies, Foster City, CA, USA). 

### 4.4. Alkaloid Standards

The following reference compounds were obtained from Sigma-Aldrich (St. Louis, MO, USA): ergonovine maleate, α-ergocryptine and ergotamine D-tartrate. Ergosedmine had been isolated and purified at the Norwegian Veterinary Institute, while erginine, ergosine/ergosinine, ergovaline, ergocornine/ergocorninine and ergocristine/ergocristinine were gifts from Dr. Michael Sulyok (IFA-Tulln, University of Natural Resources and Applied Life Sciences, Tulln, Austria). Other ergopeptine (ergogaline) and ergopeptide lactam alkaloids (ergocornam, ergocryptam, ergocristam and ergosedmam) had been studied in previous projects and were tentatively identified in *C. purpurea* sclerotia based on their mass spectral characteristics [46]. The recently explored indole-diterpene diversity in *C. purpurea* sclerotia has tentatively characterized different analogues in sclerotia from *Phalaris arundinacea* [20].

### 4.5. Alkaloid Analyses 

Sclerotium halves from the identical season, plot, and grass species, as well as genetic group (typically consisting of 10–20 sclerotium halves) were pooled and homogenized (Ultra-Turrax T25, Janke and Kunkel, Staufen im Breisgau, Germany) with 1–4 mL acetone/water (4:1, v/v; acetone of HPLC quality, Romil Ltd., Cambridge, UK), depending on the available amount of sclerotia. The homogenates were left to sediment for 30 min, and 1 mL of the supernatant was filtered through 0.22 μm Nylon membranes (Spin-X, Costar, Corning Inc, Corning, NY, USA), and transferred to chromatography vials. For ergot alkaloid analysis, 5 μL aliquots of the extracts were injected into an HPLC-ion trap mass spectrometer, consisting of a Finningan Surveyor MS Pump Plus and Autosampler Plus, a Surveyor PDA Plus photodiode array detector and Finnigan LTQ linear ion trap mass spectrometer (Thermo Fisher Scientific Inc., Waltham, MA, USA). The mass spectrometer was equipped with an electrospray ionization interface (ESI) operated in the positive mode. Separation of ergot alkaloids was either achieved on a 50 × 2.1 mm i.d. SunFire C_18_ column (3 µm particles; Waters, Milford, MA, USA), or a 50 × 2.1 mm i.d. Kinetex C_18_-XB column (2.6 µm particles; Phenomenex, Torrance, CA, USA), using a mobile phase consisting of 2 mM ammonium carbonate in water (A) and acetonitrile (B) at a flow rate of 300 μL/min. A linear gradient was applied to the column starting with 20% B to 97% B over 15 min. The column was then flushed with 97% A for 2 min, before returning to starting conditions. Important parameters for the ESI interface were a capillary temperature at 300 °C, a sheath gas flow at 40 units (approximately 40 L/min), an auxiliary gas flow at 15 units (approximately 15 L/min), and a source voltage of 5 kV. The capillary voltage and the tube lens offset were tuned by infusion of a solution of 5 μg/mL of ergovaline in methanol prior to analysis. 

For the tentative analysis of indole-diterpenes an atmospheric pressure chemical ionization (APCI) interface was used and operated in the positive mode. Separation of indole-diterpenes was achieved using a 100 × 4.6 mm i.d. Kinetex C_18_-XB column (2.6 µm particles; Phenomenex), using a mobile phase consisting of water (A) and acetonitrile/water (97.5:2.5, *v*/*v*; B), both containing 2 mM ammonium formate and 2 mM formic acid, with a flow rate of 700 μL/min. A linear gradient was applied to the column starting with 50% B to 100% B over 12 min. The column was then flushed with B for 2 min, before returning to the starting conditions. The parameters for the APCI interface were: a vaporizer temperature at 410 °C, a capillary temperature at 300 °C, a sheath gas flow at 55 units, an auxiliary gas flow at 20 units, and a corona discharge voltage of 6.0 kV. The capillary voltage and tube lens offset were tuned by infusion of a 5 μg/mL solution of paxilline in methanol. The mass spectrometer was run in the full-scan mode (*m/z* range for ergot alkaloids was 200–700 and *m/z* range for indole-diterpenes was 350–800), and with data-dependent scanning (performing MS^2^ of a maximum of three ions per full scan with a threshold intensity of 10^4^). The collision energy for data-dependent scanning was set to 35 units, the activation Q to 0.25, and the activation time to 30 ms. UV-Vis spectra of individual compounds were acquired by scanning the PDA from 190–450 nm.

Results from the instrumental analyses were processed using Xcalibur version 2.0.7 (Thermo Fisher Scientific). Individual indole-diterpenes were annotated according to the *m/z* ratio of their putative [M+H]^+^ ions and elution order. For example, the indole-diterpene compound affording putative [M+H]^+^ ions with *m/z* 422 eluting first from the column was annotated as “*m/z* 422 I”. For generation of (peptide) ergot alkaloid profiles the peak areas of individual analogues were obtained from the extracted ion chromatograms corresponding to their [M+H]^+^ ions, and the relative concentrations obtained by dividing with the total peak area from an extracted ion chromatogram corresponding to *m/z* 220–630 as the ion count in this mass window was almost exclusively due to ergot alkaloids. The approach for generation of indole-diterpene profiles was different. Again, the peak areas of individual analogues were obtained from the extracted ion chromatograms corresponding to their [M+H]^+^ ions. However, in order to normalize the profiles in individual extracts the peak areas were divided by the mass of the extracted sclerotia. Variations in the volume of extraction solvent were accounted for by including dilution factors in the calculations.

### 4.6. Bioinformatics and Statistical Analyses 

The sequences were aligned in BioEdit v.7.1.3 [47], and ITS and corresponding *tubB* sequences were combined in a 924 position alignment. The phylogenetic analyses (parsimony) were performed in MEGA *vs.*5 [48] on the combined ITS and *tubB* data set. We included sequences originating from a selection of 28 sclerotia of different hosts and habitats of this study where both ITS and corresponding *tubB* sequences had been generated. All recorded ITS and *tubB* sequence types were represented. We included a total of five reference strains of *C. purpurea sensu lato* (G2 strain WHS; ITS: EU559018, *tubB*: EU558997, *C. arundinis* strain CCC480; ITS: EU344983, *tubB*: JX083420, G3 strain ARG1; ITS: JX083542, *tubB*: JX083473, G3 strain CPE10; ITS: EU559007, *tubB*: EU558986, and G1 strain WFA; ITS: EU559017, *tubB*: EU558996). *Claviceps grothii* (CBS isolate 124.47; ITS: AJ133395, *tubB*: FJ711480) was used as the outgroup. The information given by the presence of indels (gaps) was considered to be of importance and we therefore selected parsimony analysis scoring gaps as fifth state. Indels were scored so that multi-position gaps only counted as one evolutionary event. The maximum parsimonious tree was obtained using the tree bisection and regrafting (TBR) algorithm [49] with search level 1 in which the initial trees were obtained by the random addition of sequences (10 replicates), and by bootstrap with 1000 replicates. Minimum spanning (haplotype) networks were constructed separately for ITS and *tubB* using Arlequin with default settings [50], and manually drawn in Adobe Illustrator CS5. In these analyses, all sequence data were included. In both the phylogenetic and the haplotype network analyses, reference sequences from EMBL, representing the genetic groups G1–G3, were included.

All statistical analyses were conducted in R v. 3.0.2 and 3.1.1 [51] using the vegan package [52]. The major variance patterns in the alkaloid distributions were analyzed by principal component analysis (PCA). Rows of the indole-alkaloids by sample matrix contained relative alkaloid peak areas. The rows of the sample matrix were normalized to unit sum, whereas the columns were not normalized. The sample matrix was fourth root transformed to make distributions more symmetrical (log transformation could not be used due to high frequency of zeros in the data set). Relationships between principal components and explanatory factor variables (genetic group, host and habitat) were investigated by multivariate permutation tests on Euclidean distance matrices (PERMANOVA; [53]) as implemented by the adonis function in the vegan package for R [52]. Procrustes tests showed high similarity between PCAs from untransformed, as well as square root and fourth root transformed columns, with the latter being chosen due to better readability. None of the ordination diagrams showed any arch effect, which is a well-known artefact of PCA ordinations of data sets with many zeros [54].

To supplement the PCA analysis, box plots were generated with focus on a selection of the ergot alkaloids that dominated in the G1 genetic group. One-way ANOVA was used to test any relationship between indole-alkaloid content in G1, and host plant identity. ANOVA with only 2 groups are presented as the equivalent t-tests. Fisher's exact test for count data was used to test the relationship between host or habitat and the frequency of floating or sinking sclerotia within G2.

## 5. Conclusions

The genetic, chemical and ecological data of this study strongly support the recently proposed delimitation of four different biological species from the *C. purpurea sensu lato* complex, and genetic group (= species) was the most parsimonious predictor of *Claviceps* alkaloid composition. Our data did not support habitat (dry, wet, saline) as an explanatory variable of the *Claviceps* species segregation, perhaps with the exception of very wet habitats. The wet-adapted species *C. humidiphila* (= genetic group G2), was also abundant in dry and saline habitats, and in contrast to previous reports a large proportion of G2 sclerotia sank in water. Within *C. purpurea sensu lato* we identified a genetic group (G4), new to Norway, with clear preference for the grass species *Molinia caerula*, which primarily grows in very wet habitats. The G4 appeared conspecific with the recently described *C. arundinis* on *Phragmites* and *Molinia*, and is most closely related to *C. humidiphila* (G2). They share the ability to produce indole-diterpenes of the paspaline type and other still unidentified indole-diterpenes. The indole-alkaloid content of *C. purpurea sensu stricto* (G1) sclerotia produced on infected *A. arenaria* florets differed from all other G1 sclerotia analyzed in that they included indole-diterpenes that were generally absent in G1 from all other hosts studied. The results call for a thorough genetic and chemical exploration of indole-diterpene evolution and production in *C. humidiphila* (G2) and *C. arundinis* (G4) in particular, and also in *C. purpurea* (G1) originating from *A. arenaria*.

## Supplementary Materials

Supplementary materials can be accessed at: http://www.mdpi.com/2072-6651/7/5/1431/s1.
