# Supplementary material for: Links between Genetic Groups, Indole Alkaloid Profiles and Ecology within the Grass-Parasitic Claviceps purpurea Species Complex
Source: Toxins (Basel). 2015 Apr 28;7(5):1431–56. doi: 10.3390/toxins7051431 (PMC4448156; doi:10.3390/toxins7051431)
Supplement: Supplementary file 1 [file toxins-07-01431-s001.pdf]

Supplementary Information

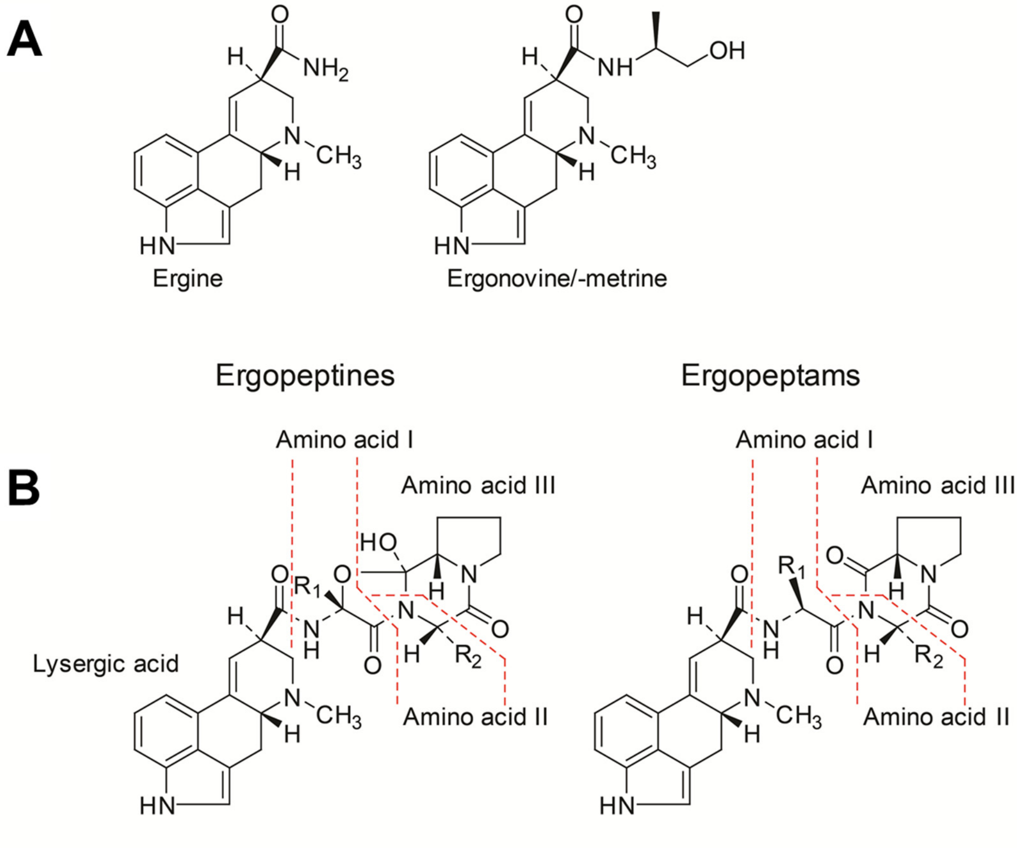

	Amino acid I	R_1
Ergotamine group	L-alanine	CH_3
Ergotoxine group	L-valine	$CH(CH_3)_2$
Ergoxine group	L-2-aminobutyric acid	CH_2CH_3
β-ergoannine group	L-isoleucine	$CH(CH_3)CH_2CH_3$

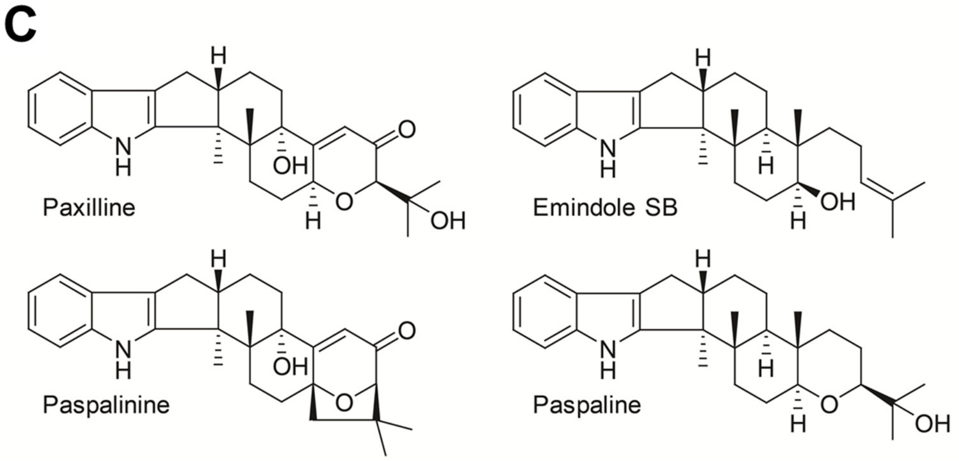

Figure S1. Examples of structures for ergot alkaloids and indole-diterpenes: ergine and ergonovine (**A**), ergopeptines and ergopeptams (**B**), examples of *Claviceps*-related indole-diterpenes (**C**).

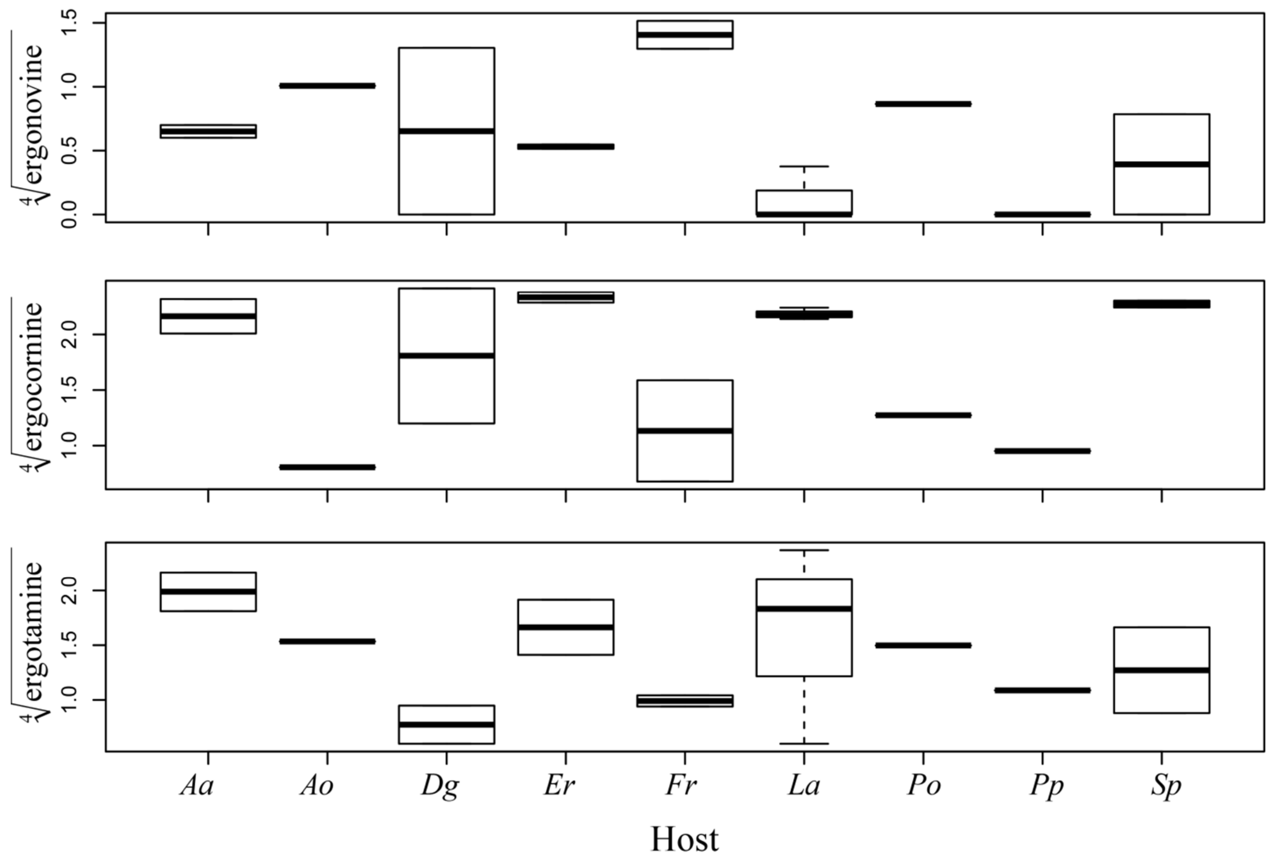

Figure S2. Differences in ergot-alkaloid composition in *Claviceps purpurea sensu stricto* (G1) sclerotia from different hosts. Substantial (but not significant) differences in relative amounts between hosts were observed for ergonovine, ergocornine and ergotamine. Host abbreviations: *Aa* = *Ammophila arenaria* *Ao* = *Anthoxanthum odoratum*, *Dg* = *Dactylis glomerata*, *Er* = *Elymus repens*, *Fr* = *Festuca rubra*, *La* = *Leymus arenarius*, *Po* = *Poa pratensis*, *Pp* = *Phleum pretense*, *Sp* = *Schedonorus pratensis*.
